# Genomic analysis of the zooplankton-associated pathogenic bacterium Spirobacillus cienkowskii reveals its functional and metabolic capacities

**DOI:** 10.1099/mgen.0.001463

**Published:** 2025-08-04

**Authors:** Pascal Angst, Alix Thivolle, Zoe Haden, Nina Wale, Dieter Ebert

**Affiliations:** 1 Department of Environmental Sciences, Zoology, University of Basel, Basel, Switzerland; 2 Department of Microbiology & Molecular Genetics, Michigan State University, East Lansing, Michigan, USA; 3 Department of Integrative Biology, Michigan State University, East Lansing, Michigan, USA; 4 Program in Ecology, Evolution and Behavior, Michigan State University, East Lansing, Michigan, USA

**Keywords:** *Daphnia*, genome assembly, Oxford Nanopore Technologies (ONT) duplex, parasite, *Silvanigrellales*

## Abstract

Genomic information can yield new insights into the molecular and physiological mechanisms that underpin pathogen virulence and transmission. We decode the genome of *Spirobacillus cienkowskii* Metchnikoff 1889, a gram-negative bacterium and one of the first described parasites of *Daphnia*. We use long-read sequencing and extensive annotation to assemble the complete circular genome of 2.81 Mbp with 2,486 protein-coding genes. In addition to antiviral systems, including CRISPR-Cas and restriction-modification systems, we describe the likely molecular basis of the unusual red phenotype of *S. cienkowskii*, which results from carotenoid production. We further describe genetic modules that may mediate this bacterium’s interactions with its host and environment. Our study provides insight into the metabolic and functional capacities of a parasite through the assessment of its genome. More generally, it demonstrates what can be learnt by applying recent advances in high-throughput sequencing to the study of parasites.

**Impact Statement**

*Spirobacillus cienkowskii* is a long-known parasite of *Daphnia*, discovered by Nobel laureate Élie Metchnikoff in 1889, and is emerging as a model system for host–parasite interactions. With the advent of high-throughput sequencing, it has become easier than ever to access the genetic basis of its striking traits like carotenoid production, which holds significance for both industrial and medical purposes. Our analysis of the *S. cienkowskii* genome demonstrates the application of the latest long-read sequencing and annotation techniques for unravelling the metabolic and functional capacities, which will push our understanding of parasite ecology and evolution.

## Data Summary

Genomic reads are deposited at the NCBI SRA database; the assembled genome, as well as the predicted set of protein sequences, is available at the NCBI GenBank database (BioProject ID: PRJNA1081344); and bioinformatic code is available at https://github.com/pascalangst/Angst_etal_2025_MicrobGenom/.

## Introduction

**Fig. 1.**
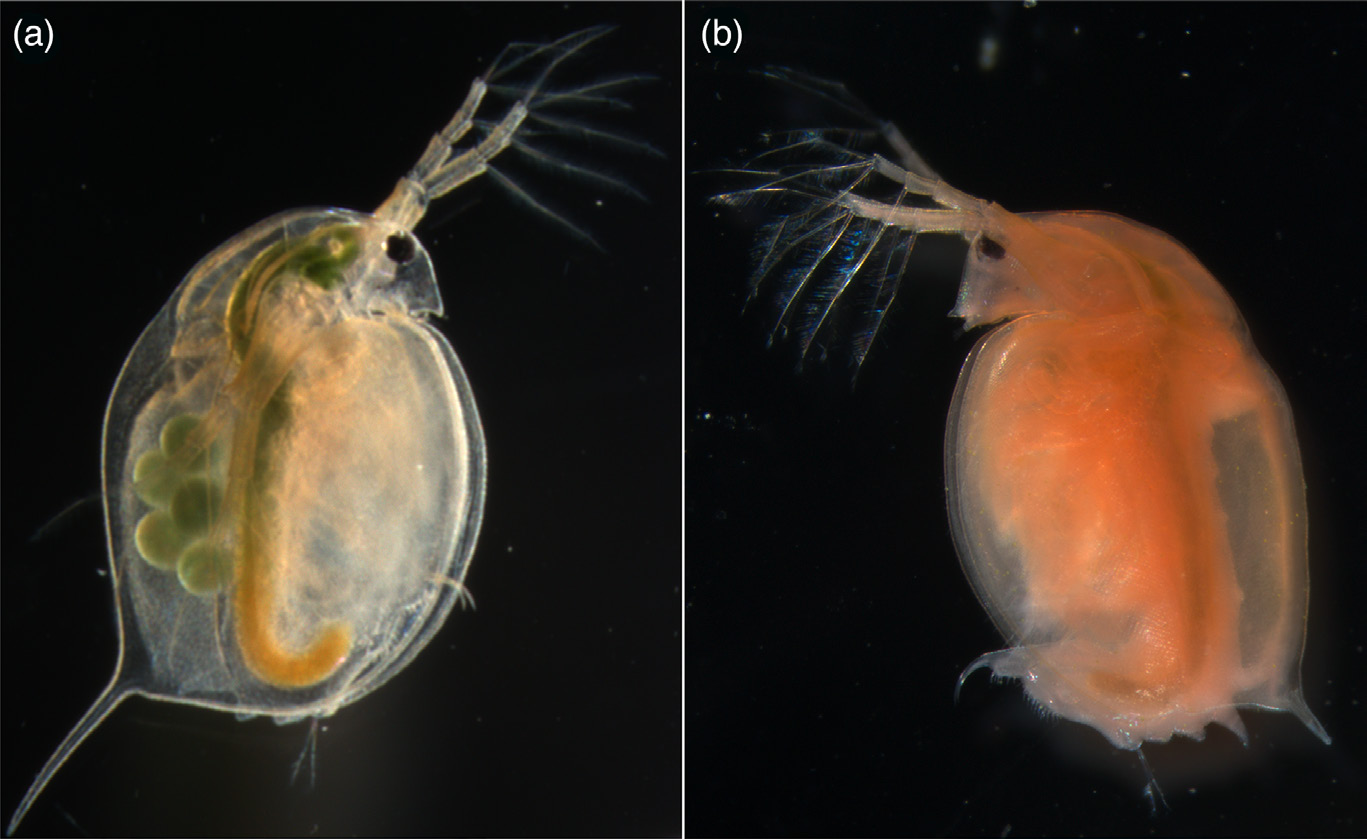
Microscope images of (un)infected *Daphnia magna*. Images show a healthy *D. magna* (**a**) and a *D. magna* naturally infected with *S. cienkowskii* (**b**). *S. cienkowskii* mainly infects the haemolymph of the host, which turns yellow to red due to the carotenoid production of *S. cienkowskii*. Both *D. magna* were collected from rock pools in the Tvaerminne archipelago and have a body length of ~3–4 mm.

Several parasites of the planktonic freshwater crustacean *Daphnia* stand out as model systems in parasitology [1]. While several of these parasites have been well described, detailed study of the biology and molecular characteristics of some parasites has remained difficult because they are hard to study in the laboratory. Recent advances in molecular technologies enable us to decode full genomes, unravelling the molecular blueprints for parasite metabolic and functional capacities that underlie their complex life strategies, including interactions with their hosts [2]. Among the earliest described parasites of *Daphnia* is *Spirobacillus cienkowskii* [3], a gram-negative bacterium belonging to the class *Oligoflexia* and order *Silvanigrellales* [4,5]. *S. cienkowskii* is a highly virulent parasite of *Daphnia*, causing large epidemics and increased susceptibility to predation [6,7]. Infected hosts turn yellow to bright red, caused by carotenoid production of the bacteria (Fig. 1) [8,9]. The bacterium has a rather unusual coiled or helical cell morph [4]. The unusual pigmentation and cell morphology are shared with other members of the *Silvanigrellales*, e.g. *Silvanigrella aquatica* [5]. Although a draft genome of *S. cienkowskii* has been published, it remains unclosed [10]. Here, we present the complete genome of *S. cienkowskii* with functional annotation as a basis for understanding its pathogenicity, transmission dynamics and adaptive strategies.

## Methods

### Sample preparation and sequencing

Approximately 30 *D. magna* individuals infected with *S. cienkowskii* were collected on 11 September 2023 from four neighbouring rock pools on the island Spicarna in the Tvaerminne archipelago, southwestern Finland (59° 48′ 43.0″ N 23° 12′ 30.8″ E). Infected animals, recognizable by their bright red colour, were collected from the rock pools with long wide-mouth pipettes for DNA extraction and molecular diagnostics. They were transported to the laboratory, where they were placed in RNAlater (Ambion, Glasgow, UK) and kept cold until DNA isolation. We obtained high-molecular-weight DNA from host and parasite using the GenePure DNA Isolation Kit (QIAGEN, Hilden, Germany) as described in Angst *et al*. [11] with the addition of a 70 µm mesh (pluriSelect, Leipzig, Germany) for filtering undigested tissue after Proteinase K treatment (https://dx.doi.org/10.17504/protocols.io.5jyl82n96l2w/v1 [12]). From this, an Oxford Nanopore Technologies (Oxford, UK) genomic DNA library was prepared with the SQK-LSK114 ligation kit and sequenced on a MinION device with a Spot-ON Flow Cell (R10.4.1).

### Genome assembly and annotation

The obtained sequencing reads (total/N50 : 8.90 Gb/1.40 kb) were basecalled with dorado v.0.3.4 (https://github.com/nanoporetech/dorado) in duplex mode using the dna_r10.4.1_e8.2_400bps_sup@v4.2.0 model, converted to FASTQ format using BEDTools v.2.30.0 [13] and then assembled with Flye v.2.9.2-b1786 [14] in meta mode. Unless otherwise stated, we used default parameters for bioinformatics software. We extracted the longest contig from the assembly, which was circular and had the highest coverage of all contigs, oriented it according to the replication origin identified using OriFinder v.2022 [15] and checked its completeness using BUSCO v.5.4.5 [16] with its proteobacteria_odb10 database (creation date: 23 February 2021), which encompasses 219 genes. Other circular contigs were very short, had low coverage and showed no signatures of plasmid origin. We annotated the assembly using Bakta v.1.8.2 [17] with its Bakta database v.5 and additionally identified transposable elements using TransposonUltimate v.1.0 [18], antiviral systems using CRISPRCasTyper v.1.8.0 [19] and DefenseFinder v.1.2.0 [20] and prophages using PHASTER [21]. We inferred the metabolic function of genes using BlastKOALA v.3.0 [22] and DRAM (Distilled and Refined Annotation of Metabolism) v.1.2.0 [23] software. Genomic features were plotted using R v.4.2.2 [24] packages circlize v.0.4.15 [25] and ComplexHeatmap v.2.14.0 [26].

### Sequence comparison within *S. cienkowskii*

For within-species comparison, we downloaded the previously generated draft assembly of *S. cienkowskii* and its annotated gene sequences from NCBI GenBank (NCBI database; assembly name: ASM333960v1; GenBank assembly accession: GCA_003339605.1; BioProject accession: PRJNA450308; Bresciani *et al*. [10]). We used Proteinortho v.6.1.7 [27] to identify single-copy orthologues and aligned them using prank v.170427 [28] as described in Fields *et al*. [29], whereby we used the R package seqinr v.4.2–36 [30] for file handling. We calculated average nucleotide identity between the assemblies using OrthoANI v.0.93.1 [31] and nucleotide diversity of genes from the separate alignments using the script selectionStats.py (https://github.com/tatumdmortimer/popgen-stats).

## Results and discussion

**Fig. 2.**
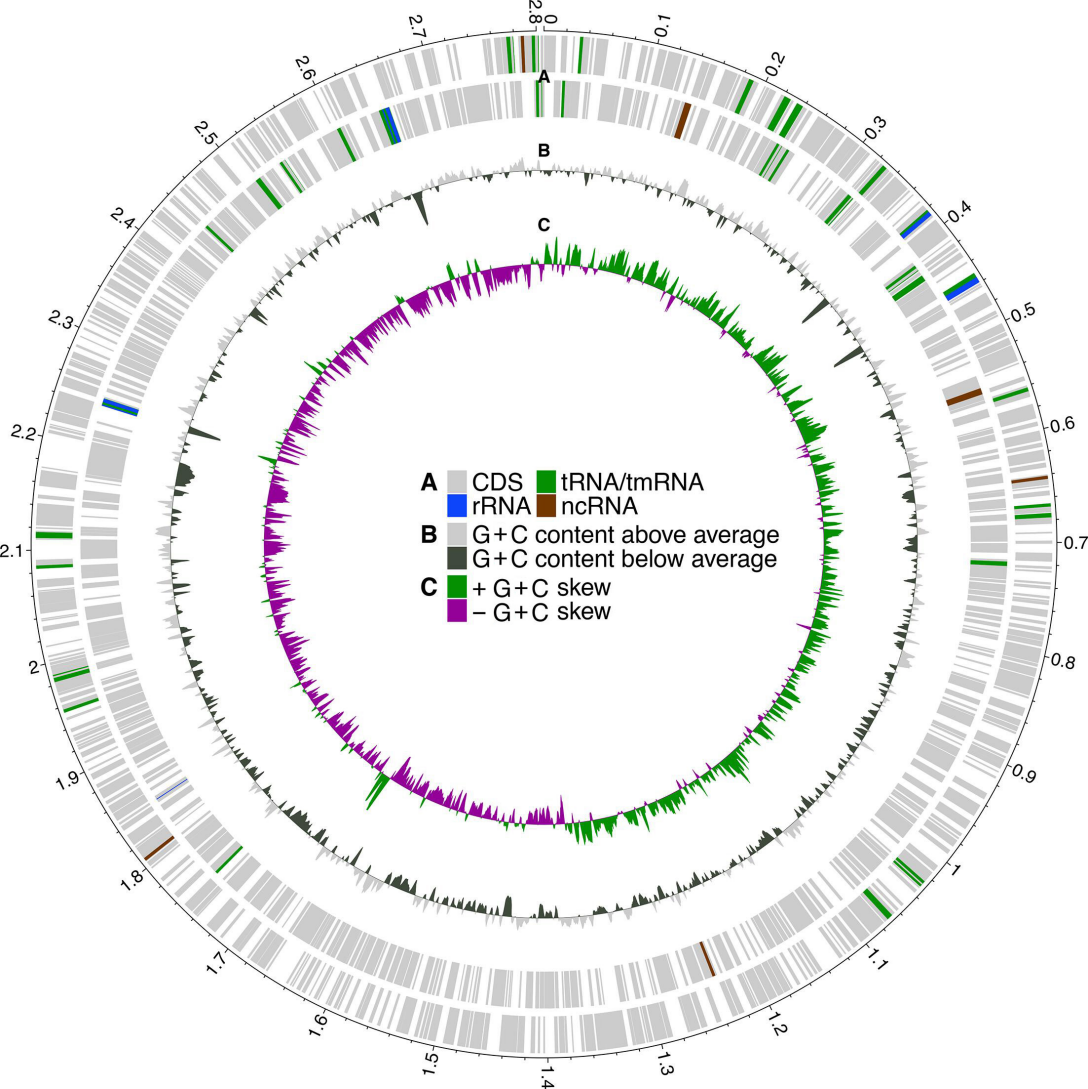
Circular map showing genomic features of the *S. cienkowskii* genome. (A) Genomic features plotted on the forward and reverse strands. Non-CDS features are enlarged for better visibility. (B) G+C content per sliding window across the genome above (outside the circle) and below (inside the circle) the average G+C ranges from −0.20 to 0.12. (C) G+C skew greater than 0 (guanine overrepresentation; outside the circle) and less than 0 (guanine underrepresentation; inside the circle) ranges from −0.26 to 0.35. The genome is oriented from the replication origin, and the circular scale on the outside is in Mbp. The broad peak in G+C content just below 2.2 Mbp without features on the forward strand corresponds to a cluster of ribosomal proteins.

Genome assembly of *S. cienkowskii* resulted in a single circular contig with a length of 2.81 Mbp and a G+C content of 32.19% (Fig. 2, Table 1). The assembly contained 2,486 protein-coding genes with a mean length of 1,017 bp, covering 90.98% of the genome (Table 1). The BUSCO score of the assembly as compared to the Proteobacteria BUSCO reference set was 74% (73.5% complete, single-copy plus 0.5% complete, duplicated). All of these genomic features are similar in other representative genomes of the *Silvanigrellales*, within which *S. cienkowskii* is most closely related to the genus *Pigmentibacter* according to previous phylogenomic analysis [32]. For example, BUSCO scores of the other complete genomes are 75.8% for *S. aquatica* [5], 77.2% for *Fluviispira sanaruensis* [33] and 76.8% for *Pigmentibacter* sp. JX0631 (NCBI database; assembly name: ASM2987325v1; GenBank assembly accession: GCA_029873255.1; BioProject accession: PRJNA955423). The low BUSCO scores, a common measure of assembly completeness, of the complete *Silvanigrellales* genomes suggest that the Proteobacteria BUSCO reference set is not optimized for this clade. The related genomes have plasmids and a CRISPR-Cas Type I system [5,33]. Our genome assembly of *S. cienkowskii* did not result in any circular contigs of similar length to the plasmids of the related species, suggesting that this parasite is unlikely to have plasmids. We found eight antiviral systems including each a CRISPR-Cas Type I-F and II-C system, four restriction-modification systems, a SoFic system and a Gabija system (Table S1, available in the online Supplementary Material). We also found 61 transposable elements (51 DNA transposons, 9 retrotransposons and 1 unclassified) and 1 prophage (Table S1).

**Table 1.** Statistics of the assembly and annotations of the *S. cienkowskii* genome

Assembly	
Total length (Mbp)	2.81
Coverage	1,745×
G+C content (%)	32.19
BUSCO completeness score (%)	74

Annotation	
Number of mRNAs	2,486
Number of ncRNAs	6
Number of rRNAs	13
Number of tmRNAs	1
Number of tRNAs	42

To elucidate the metabolic potential of *S. cienkowskii*, we functionally annotated the predicted genes. Thereby, we found a pathway to synthesize the red carotenoid lycopene from glucose or other metabolites (Table S2). Some genes of this pathway have been previously reported in *S. cienkowskii* [10]. Specifically, the identified pathway includes glycolysis with pyruvate oxidation, the mevalonate pathway to synthesize the terpenoid backbone of carotenoids and carotenoid synthesis via phytoene/squalene synthetase and phytoene desaturase. We further found a glycogen phosphorylase gene (Table S2). This enzyme could potentially be secreted by *S. cienkowskii* to break up host glycogen and hence increase glucose availability in the host haemolymph, which it infects. Red pigmentation is a phenotypic characteristic to distinguish *S. cienkowskii* from other *Daphnia*-infecting parasites [8], but genes for glycolysis, the mevalonate pathway and lycopene synthesis also exist in the non-*Daphnia*-infecting relative *S. aquatica* [5]. Since the *Silvanigrellales* are all pigmented, it is unlikely that pigmentation represents an adaptation to a parasitic lifestyle. However, carotenoid production may nonetheless be beneficial in this context, as it may help the parasite to quench reactive oxygen species produced by the host immune system [9,34].

Comparison with the *S. cienkowskii* draft assembly generated from the haemolymph of a North American *Daphnia dentifera* genotype [10] allowed us to analyse nucleotide diversity at the gene level. The genes involved in the carotenoid synthesis pathway had low nucleotide diversity compared to, for example, BUSCO genes (Fig. S1). Contrarily, the three chitinases detected with the DRAM software (gene IDs: Spiro2_02780, Spiro2_04520 and Spiro2_04605) showed relatively high nucleotide diversity (Fig. S1). This suggests that, while the carotenoid pathway may be under purifying selection, the chitinases experience diversifying selection. Chitinases could facilitate the degradation of chitin-rich structures such as *Daphnia*’s carapace and the shell of their resting eggs, which also become infected [7,35], into monosaccharides. Enzymes may thus be important for the invasion or escape of the host structures that support * S. cienkowskii*’s growth. Monosaccharides could further be used in the glycolysis of *S. cienkowskii* via glucosamine import. Interpretation of nucleotide diversity was difficult for genes with unknown function, i.e. hypothetical proteins. This was the case for some genes with very high estimates of nucleotide diversity (Table S3). We detected a cluster of 21 genes encoding hypothetical proteins in a small region of the *S. cienkowskii* genome (1,664–1,678 kbp) with a total of 23 genes that diverged strongly in G+C skew (Fig. 2). High within-species nucleotide diversity of genes and the inability to functionally annotate them suggests rapid evolution in this relatively understudied taxon, with a possible history of horizontal gene flow for genes in regions of divergent G+C skew.

Finally, we found both low- and high-affinity type IV complexes of the electron transport chain, i.e., the cytochrome c oxidase and the cytochrome bd ubiquinol oxidase complex (Fig. S2). This suggests that *S. cienkowskii* is flexible in its oxygen-use capacities and may be able to tolerate low-oxygen environments [36]. This is consistent with the idea that *S. cienkowskii* aggregates in such environments (e.g. at the sediment–water interface of lakes), where it might occur independently of the host or after its host died and sank to the ground [35,37]. A motile free-living stage has been proposed for the transmission of *S. cienkowskii* [7,10], and motile stages have been observed in cultures of other *Silvanigrellales* [38]. Motility might be achieved by a flagellum, for which we found all structural genes common to bacteria with a functional flagellum [39,40]. In addition, we found all the underlying genes of the chemotaxis pathway in *Escherichia coli* in our assembly [41], suggesting that chemotaxis mechanisms may determine the direction of *S. cienkowskii*’s movement. The genome of *S. cienkowskii* is not as reduced as that of other pathogenic bacteria [42] and contains modules essential for free-living bacteria, including glycolysis, the citrate cycle and the biosynthesis of fatty acids, nucleotides and some amino acids (Fig. S2). This aligns with the recent notion that *S. cienkowskii* may spend part of its life cycle outside of the host [7].

Our comparisons of the new complete genome assembly with the previous *S. cienkowskii* draft assembly were limited to small-scale genetic variation, i.e. SNPs (Fig. S1). The variation is low (OrthoANI value: 99.07%) and suggests that they represent the same species. Large-scale structural variation among them is difficult to assess, as the draft assembly of the parasite obtained from * D. dentifera* consists of many small contigs. For example, we found four copies of the 16S–23S rRNA genes, whereas Bresciani *et al*. [10] described one. The variation in gene copy number among the two assemblies is likely due to assembly problems of the non-contiguous draft genome. Our assembly adds to the scarce resources available for the order *Silvanigrellales*, of which only a few members are described and only three other genomes have been fully sequenced (*S. aquatica*, *F. sanaruensis* and *Pigmentibacter* sp. JX0631). Comparative genomics across *Silvanigrellales* and beyond will help to understand the genetic basis of (parasitic) traits and their ecology and evolution. Given the industrial and medical interest in the diverse pigment production of *Silvanigrellales*, such comparisons might inform applied research [43,44]. A better understanding of the biosynthesis of biodegradable and environmentally friendly microbial pigments will advance the replacement of synthetic pigments, reducing their negative impacts on the environment and human health.

## Emended description of *Spirobacillus cienkowskii* Metchnikoff 1889

*Spirobacillus* (Spi.ro.ba.ci'llus. Gr. fem. n. speîra, coil or spiral, referring to the coil shape of this organism; L. masc. n. bacillus, cylindrical or rod-like bacterium; N.L. masc. n. Spirobacillus, rod-like bacterium with a coil shape).

Nomenclatural type species of the genus: *Spirobacillus cienkowskii* [ci.en.kow.ski'i. N.L. gen. n. cienkowskii, in memory of Lev Semyonovich Tsenkovsky (Leon Cienkowski), a Polish-Ukrainian botanist, protozoologist and bacteriologist, for his contributions to the theory of pleomorphism].

The descriptions were initially provided by Metchnikoff [3] and later adopted by Rodrigues *et al*. [4]. *S. cienkowskii* is the first described member of the family *Silvanigrellaceae* [38], Proteobacteria of the class *Oligoflexia* and the order *Silvanigrellales*. It forms coils (3.50 µm long by 1.07 µm wide) and produces a pink carotenoid. It is known to parasitize the haemolymph of several species of *Daphnia*. The whole-genome sequence is available from NCBI GenBank under the accession number GCA_037081835.1. The SeqCode accession is seqco.de/r:hyd0fmmd.

## Supplementary material

10.1099/mgen.0.001463 Uncited Supplementary Material 1.

## Abbreviations

DRAM: Distilled and Refined Annotation of Metabolism
