# Supplementary material for: Genomic analysis of the zooplankton-associated pathogenic bacterium Spirobacillus cienkowskii reveals its functional and metabolic capacities
Source: Microb Genom. 2025 Aug 4;11(8):001463. doi: 10.1099/mgen.0.001463 (PMC12321486; doi:10.1099/mgen.0.001463)
Supplement: Uncited Supplementary Material 1. [file mgen-11-01463-s001.pdf]

Supplemental Information for:

Genomic analysis of the zooplankton-associated pathogenic bacterium Spirobacillus cienkowskii reveals its functional and metabolic capacities

Pascal Angst[1], Alix Thivolle[1], Zoe Haden[2], Nina Wale[2,3,4,*], and Dieter Ebert[1]

[1] Department of Environmental Sciences, Zoology, University of Basel, Basel, Switzerland
[2] Department of Microbiology & Molecular Genetics, Michigan State University, East Lansing, Michigan, USA
[3] Department of Integrative Biology, Michigan State University, East Lansing, Michigan, USA
[4] Program in Ecology, Evolution and Behavior, Michigan State University, East Lansing, Michigan, USA
[*] Corresponding author: walenina@msu.edu

Figure S1: Ratio of nonsynonymous to synonymous nucleotide diversity (πN/πS) of *S. cienkowskii* genes. Groups of genes mentioned in the text are grouped separately. Other genes are in the "NA" group, representing the genomic background**.**

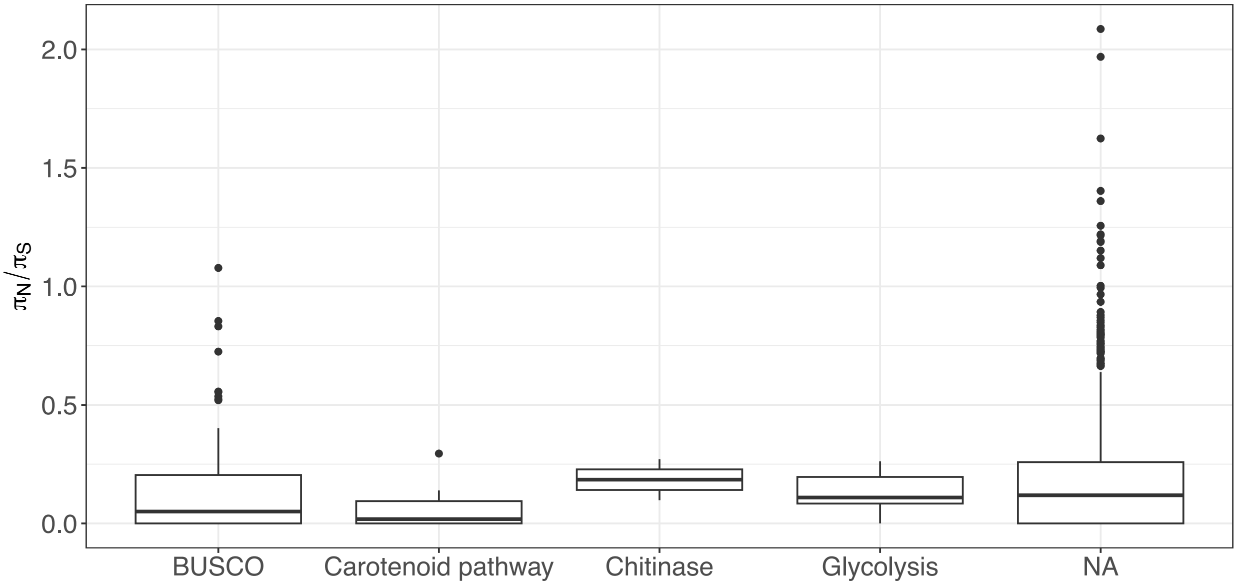

Figure S2: Summary of the analysis with the DRAM (Distilled and Refined Annotation of Metabolism) software showing traits likely exhibited by *S. cienkowskii*. The heatmap on the left shows the presence of certain annotations, indicative of the presence of characteristic metabolic functions. The heatmap on the right gives the completeness of various electron transport chain (ETC) complexes and other pathways representing common, relevant metabolisms. Colors indicate the presence/absence or completeness of the respective feature.

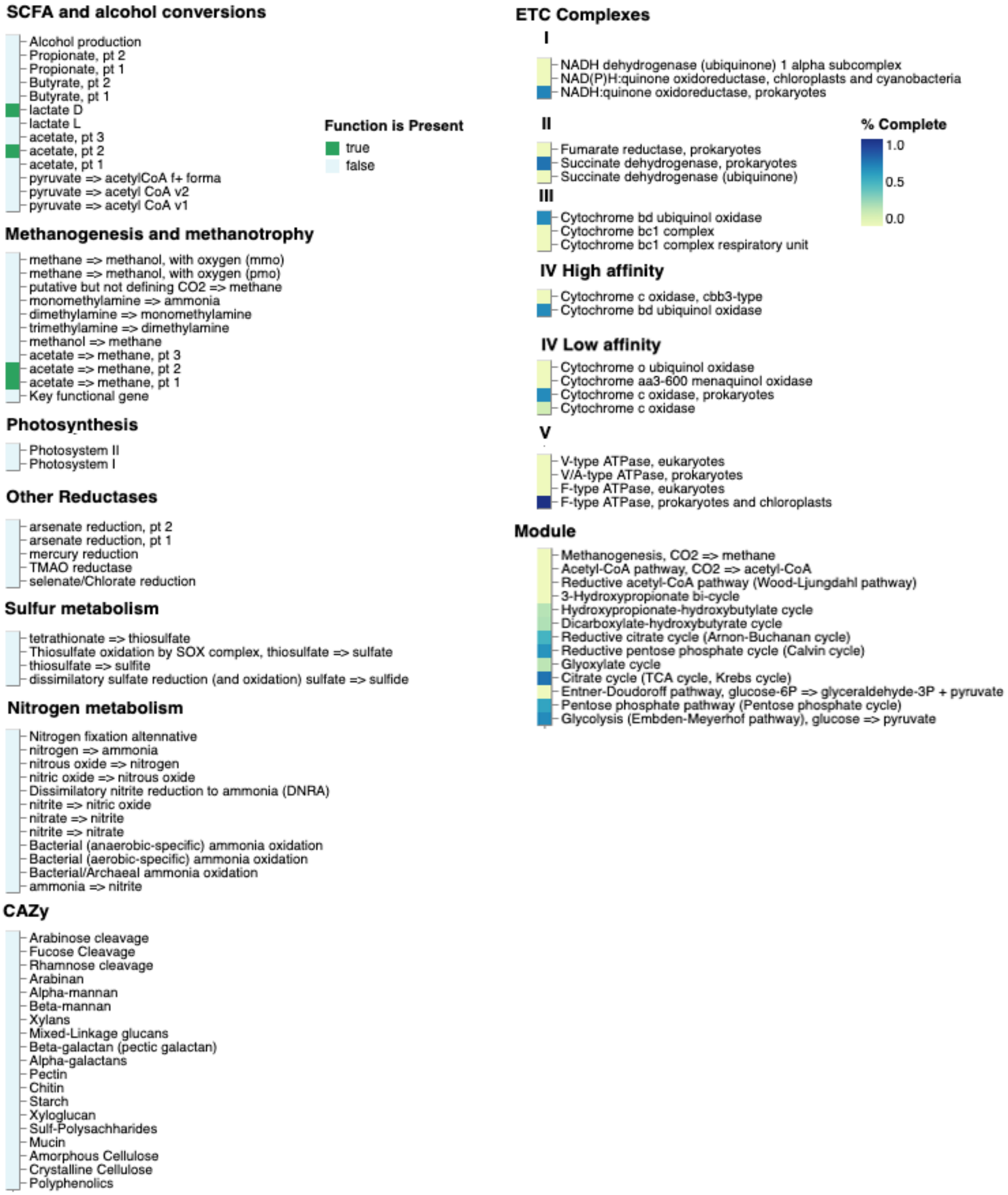

Table S1: Feature annotation including transposons, antiviral systems, and a prophage.

Start position	End position	Feature	Number of genes	Detection software
24,115	24,355	Zator,TIR DNATransposon	NA	TransposonUltimate
45,179	46,709	Zator,TIR DNATransposon	NA	TransposonUltimate
45,342	45,508	Zator,TIR DNATransposon	NA	TransposonUltimate
45,675	45,841	Zator,TIR DNATransposon	NA	TransposonUltimate
46,008	46,174	Sola,TIR DNATransposon	NA	TransposonUltimate
46,341	46,507	Zator,TIR DNATransposon	NA	TransposonUltimate
99,465	101,607	hAT,TIR DNATransposon	NA	TransposonUltimate
126,154	126,518	Zator,TIR DNATransposon	NA	TransposonUltimate
155,361	157,459	Zator,TIR DNATransposon	NA	TransposonUltimate
297,574	298,740	SoFic	1	DefenseFinder
300,907	303,162	RM_Type_II	2	DefenseFinder
303,162	305,633	RM_Type_IIG	1	DefenseFinder
310,806	316,847	RM_Type_I	3	DefenseFinder
324,252	324,575	Sola,TIR DNATransposon	NA	TransposonUltimate
353,383	356,353	Gypsy,LTR Retrotransposon	NA	TransposonUltimate
545,837	546,493	hAT,TIR DNATransposon	NA	TransposonUltimate
590,161	590,523	Zator,TIR DNATransposon	NA	TransposonUltimate
822,628	822,827	Zator,TIR DNATransposon	NA	TransposonUltimate
824,215	824,641	Tc1-Mariner,TIR DNATransposon	NA	TransposonUltimate
1,042,703	1,043,124	Zator,TIR DNATransposon	NA	TransposonUltimate
1,082,618	1,090,870	CMC,TIR DNATransposon	NA	TransposonUltimate
1,196,201	1,196,355	Zator,TIR DNATransposon	NA	TransposonUltimate
1,283,677	1,284,184	hAT,TIR DNATransposon	NA	TransposonUltimate
1,319,482	1,329,742	Gypsy,LTR Retrotransposon	NA	TransposonUltimate
1,373,099	1,373,297	Tc1-Mariner,TIR	NA	TransposonUltimate

		DNATransposon		
1,379,124	1,382,221	Gabija	2	DefenseFinder
1,385,940	1,386,120	Zator,TIR DNATransposon	NA	TransposonUltimate
1,432,965	1,433,118	Zator,TIR DNATransposon	NA	TransposonUltimate
1,435,984	1,436,120	Zator,TIR DNATransposon	NA	TransposonUltimate
1,436,423	1,436,584	Tc1-Mariner,TIR DNATransposon	NA	TransposonUltimate
1,456,541	1,456,859	Zator,TIR DNATransposon	NA	TransposonUltimate
1,543,435	1,543,918	Zator,TIR DNATransposon	NA	TransposonUltimate
1,575,579	1,582,230	CMC,TIR DNATransposon	NA	TransposonUltimate
1,603,816	1,606,320	Helitron DNATransposon	NA	TransposonUltimate
1,630,711	1,635,082	Zator,TIR DNATransposon	NA	TransposonUltimate
1,652,839	1,654,559	Gypsy,LTR Retrotransposon	NA	TransposonUltimate
1,664,033	1,665,844	Gypsy,LTR Retrotransposon	NA	TransposonUltimate
1,673,482	1,681,020	Copia,LTR Retrotransposon	NA	TransposonUltimate
1,815,942	1,816,100	Zator,TIR DNATransposon	NA	TransposonUltimate
1,876,713	1,881,951	CRISPR-Cas II-C	3<br>9 CRISPR repeats<br>1 anti-repeat	CRISPRCasTyper
1,923,129	1,923,295	Zator,TIR DNATransposon	NA	TransposonUltimate
1,924,497	1,938,040	CMC,TIR DNATransposon	NA	TransposonUltimate
1,992,482	1,994,471	hAT,TIR DNATransposon	NA	TransposonUltimate
1,994,795	1,996,938	Zator,TIR DNATransposon	NA	TransposonUltimate
2,033,039	2,033,391	Zator,TIR DNATransposon	NA	TransposonUltimate
2,093,363	2,095,106	Zator,TIR DNATransposon	NA	TransposonUltimate
2,113,724	2,113,851	Zator,TIR DNATransposon	NA	TransposonUltimate
2,113,979	2,116,007	RM_Type_II	2	DefenseFinder
2,222,120	2,222,338	Zator,TIR	NA	TransposonUltimate

		DNATransposon		
2,247,290	2,247,438	Zator,TIR DNATransposon	NA	TransposonUltimate
2,268,232	2,268,749	Zator,TIR DNATransposon	NA	TransposonUltimate
2,272,017	2,272,273	Zator,TIR DNATransposon	NA	TransposonUltimate
2,274,515	2,274,675	Zator,TIR DNATransposon	NA	TransposonUltimate
2,278,391	2,292,701	Prophage	18	PHASTER
2,283,964	2,288,569	Gypsy,LTR Retrotransposon	NA	TransposonUltimate
2,285,384	2,292,507	Gypsy,LTR Retrotransposon	NA	TransposonUltimate
2,294,414	2,294,556	Tc1-Mariner,TIR DNATransposon	NA	TransposonUltimate
2,327,169	2,327,729	Zator,TIR DNATransposon	NA	TransposonUltimate
2,328,483	2,337,712	CRISPR-Cas I-F	6<br>25 CRISPR repeats	CRISPRCasTyper
2,332,438	2,332,636	Zator,TIR DNATransposon	NA	TransposonUltimate
2,332,741	2,332,936	unclassified, Transposon	NA	TransposonUltimate
2,348,871	2,349,008	Zator,TIR DNATransposon	NA	TransposonUltimate
2,371,403	2,371,618	Zator,TIR DNATransposon	NA	TransposonUltimate
2,389,233	2,391,955	CMC,TIR DNATransposon	NA	TransposonUltimate
2,392,412	2,397,034	Gypsy,LTR Retrotransposon	NA	TransposonUltimate
2,397,896	2,402,530	Copia,LTR Retrotransposon	NA	TransposonUltimate
2,418,062	2,424,036	CMC,TIR DNATransposon	NA	TransposonUltimate
2,448,791	2,448,940	Zator,TIR DNATransposon	NA	TransposonUltimate
2,571,043	2,571,246	Zator,TIR DNATransposon	NA	TransposonUltimate
2,699,390	2,699,592	Sola,TIR DNATransposon	NA	TransposonUltimate

Table S2: Genes of *S. cienkowskii* putatively involved in the biosynthesis of carotenoids.

Pathway	Enzyme	EC Number	Gene ID(s)
Glycogen metabolism	Glycogen phosphorylase	EC 2.4.1.1	Spiro2_07540
Glycolysis	Phosphoglucomutase	EC 5.4.2.2	Spiro2_09460
Glycolysis	Glucose-6-phosphate isomerase	EC 5.3.1.9	Spiro2_05065
Glycolysis	ATP-dependent phosphofructokinase / Diphosphate-dependent phosphofructokinase	EC 2.7.1.11, EC 2.7.1.90	Spiro2_02510
Glycolysis	Fructose-bisphosphate aldolase, class II	EC 4.1.2.13	Spiro2_08925
Glycolysis	Glyceraldehyde 3-phosphate dehydrogenase	EC 1.2.1.12	Spiro2_05235
Glycolysis	Phosphoglycerate kinase	EC 2.7.2.3	Spiro2_08105
Glycolysis	2,3-bisphosphoglycerate-independent phosphoglycerate mutase	EC 5.4.2.12	Spiro2_03885
Glycolysis	Enolase	EC 4.2.1.11	Spiro2_10955
Glycolysis	Pyruvate kinase	EC 2.7.1.40	Spiro2_04390, Spiro2_08255
Glycolysis	Pyruvate dehydrogenase E1 component subunit alpha and beta	EC 1.2.4.1	Spiro2_04255
Glycolysis	Pyruvate dehydrogenase E2 component	EC 2.3.1.12	Spiro2_04260
Mevalonate pathway	Acetyl-CoA C-acetyltransferase	EC 2.3.1.9	Spiro2_02390, Spiro2_05315, Spiro2_09515
Mevalonate pathway	Hydroxymethylglutaryl-CoA synthase	EC 2.3.3.10-	Spiro2_09515 (?), Spiro2_12215 (?)
Mevalonate pathway	Hydroxymethylglutaryl-CoA reductase	EC 1.1.1.88	Spiro2_03345
Mevalonate pathway	Mevalonate kinase	EC 2.7.1.36	Spiro2_03330
Mevalonate pathway	Phosphomevalonate kinase	EC 2.7.4.2	Spiro2_03340
Mevalonate pathway	Diphosphomevalonate decarboxylase	EC 4.1.1.33	Spiro2_03335
Mevalonate pathway	Isopentenyl-diphosphate Delta-isomerase	EC 5.3.3.2	Spiro2_03350
Mevalonate pathway	Dimethylallyltranstransferase	EC 2.5.1.1	Spiro2_08300
Mevalonate pathway	(2E,6E)-farnesyl diphosphate synthase	EC 2.5.1.10	Spiro2_08300
Mevalonate pathway	Geranylgeranyl pyrophosphate synthase	EC 2.5.1.29	Spiro2_08300
Carotenoid Biosynthesis	Phytoene/squalene synthetase	EC 2.5.1.32	Spiro2_01195
Carotenoid Biosynthesis	Phytoene desaturase	EC 1.3.99.31	Spiro2_01200

Table S3: Genes of *S. cienkowskii* with the highest ratio of nonsynonymous to synonymous nucleotide diversity (πN/πS).

Gene ID(s)	π	πN	πS	πN/πS
Spiro2_09250	0.01465798	0.0164271	0.00787402	2.0862423
Spiro2_02445	0.01156069	0.01280512	0.00650407	1.9687875
Spiro2_06780	0.00329567	0.00358959	0.00220995	1.6242896
Spiro2_02430	0.01333333	0.01410106	0.01005025	1.4030552
Spiro2_00845	0.00540123	0.00567913	0.00417537	1.3601514
Spiro2_03960	0.26309524	0.2746004	0.21862934	1.2560089
Spiro2_01260	0.04583333	0.0474359	0.03888889	1.2197802
Spiro2_00350	0.00808081	0.0083717	0.00688468	1.21599
Spiro2_08345	0.00819672	0.00845785	0.00710059	1.1911474
Spiro2_04865	0.08119658	0.08367101	0.07044265	1.1877891
Spiro2_02380	0.00213675	0.00220143	0.00191205	1.1513484
Spiro2_04220	0.05797101	0.05927052	0.05294118	1.1195542
Spiro2_07000	0.00512821	0.00521966	0.00479233	1.0891692
Spiro2_00190	0.00270563	0.00274788	0.00254885	1.0780856
Spiro2_12720	0.01212121	0.01212938	0.01209677	1.0026954
